# Bulk Density Homogenization and Impact Initiation Characteristics of Porous PTFE/Al/W Reactive Materials

**DOI:** 10.3390/ma13102271

**Published:** 2020-05-15

**Authors:** Baoqun Geng, Haifu Wang, Qingbo Yu, Yuanfeng Zheng, Chao Ge

**Affiliations:** State Key Laboratory of Explosion Science and Technology, Beijing Institute of Technology, Beijing 100081, China; gengbq@bit.edu.cn (B.G.); wanghf@bit.edu.cn (H.W.); yuqb@bit.edu.cn (Q.Y.); zhengyf@bit.edu.cn (Y.Z.)

**Keywords:** reactive materials, PTFE/Al/W, bulk density homogenization, porous, impact initiation characteristics

## Abstract

In this research, the bulk density homogenization and impact initiation characteristics of porous PTFE/Al/W reactive materials were investigated. Cold isostatic pressed (CIPed) and hot temperature sintered (HTSed) PTFE/Al/W reactive materials of five different theoretical maximum densities were fabricated via the mixing/pressing/sintering process. Mesoscale structure characteristics of the materials fabricated under different molding pressures were compared while the effect of molding pressures on material bulk densities was analyzed as well. By using the drop weight testing system, effects of the theoretical maximum densities (TMDs), drop heights and molding pressures on the impact initiation characteristics were studied. Quantitatively, characteristic drop heights (*H*_50_) for different types of materials were obtained. The two most significant findings of this research are the density homogenization zone and the sensitivity transition zone, which would provide meaningful guides for further design and fabrication of reactive materials.

## 1. Introduction

Reactive materials denote a class of materials which are composed of two or more non-explosive solids. Generally, these materials stay inert until subjected to a sufficiently strong mechanical stimulus to undergo fast burning and explosion which releases enormous chemical energy [1,2,3,4,5,6]. The unique intensive dynamic loading induced hot spots form within received extensive attention as potential structural materials for explosives [7,8,9,10] and propellants [11,12,13]. Polytetrafluoroethylene filled by aluminum and tungsten particles (PTFE/Al/W) is a typical formula applied for current reactive materials studies [10,14]. Three mixed components are fabricated to achieve high strength and high reaction efficiency under dynamic loading via the mixing/pressing/sintering process.

Reactive materials require a wide range of bulk densities to achieve idealized damage effects on different targets. One of the traditional methods to obtain different densities is to change the formulation, for example, changing the massive ratio of inert metal (W particle) for the PTFE/Al/W reactive materials. Most researchers have considered reactive materials as a continuum with uniform bulk density [1,7,10]. Actually, the bulk densities of reactive materials will be close to their theoretical maximum density via a pressing/sintering process; however, the distinction between bulk densities and theoretical maximum density still exists due to the inner structural pore. Relevant studies considering the porous structures are mainly focused on the mechanical properties and simulations of the reactive materials with a specific bulk density [4,5,12].

However, insufficiently pressed and sintered reactive materials show different theoretical maximum densities (TMD) and discrepant material properties. Bulk densities of porous reactive materials achieve specific needs [15,16,17]. Moreover, bulk density distributions influenced by pressing and sintering processes are essential for engineering applications, such as double-layered shaped charge liner and reactive material bullet core size design [18,19].

For energetic materials, hot spots would form within microstructures due to internal energy accumulation under typical high strain rate circumstances [9]. These hot spots lead to intensive explosion and deflagration. Ignoring the crack tip induced initiation mechanism under quasi-static compression [20], reactions under dynamic compression are attributed to the shear-induced mechanism which correlates the mechanical and thermal effects [1]. For reactive materials considering internal pores introduced during fabrication, the pores collapse mechanism could generate a hot spot. When large scale deformation of the porous materials occurs under dynamic impact, the asymmetric collapse of the pores distributed in the material causes the formation of hot spots due to the local temperature rise.

For the mixing/pressing/sintering reactive materials with a wide range of bulk densities, their bulk density transition characteristics and the corresponding relationship with impact initiation sensitivities are still needed to be analyzed and discussed quantitatively.

Here, in this research, density distribution characteristics of sintered PTFE/Al/W reactive materials achieved by increasing molding pressures from 5 MPa to 150 MPa are studied. Furthermore, impact initiation characteristics of PTFE/Al/W reactive materials with the TMDs of 5.2 g/cm^3^ and 7.2 g/cm^3^ are evaluated by drop weight test and are discussed from the hot spot point of view.

## 2. Experimental

### 2.1. Materials Fabrication

In this study, cold isostatic pressed (CIPed) and hot temperature sintered (HTSed) PTFE/Al/W reactive materials of five different densities of 3.2 g/cm^3^, 4.2 g/cm^3^, 5.2 g/cm^3^, 6.2 g/cm^3^ and 7.2 g/cm^3^ were fabricated via the mixing/pressing/sintering process. Materials of the five TMDs were denoted by A, B, C, D and E, as listed in Table 1. The original average size of PTFE powder is approximately 100 µm (DuPont, type MP 1500J, Wilmington, DE, USA). The average diameters of the nitrogen atomized aluminum (FLPN 291.1) and in-process raw tungsten (FW-1-200) granules selected are 29 µm and 20 µm, respectively.

**Figure 1:**
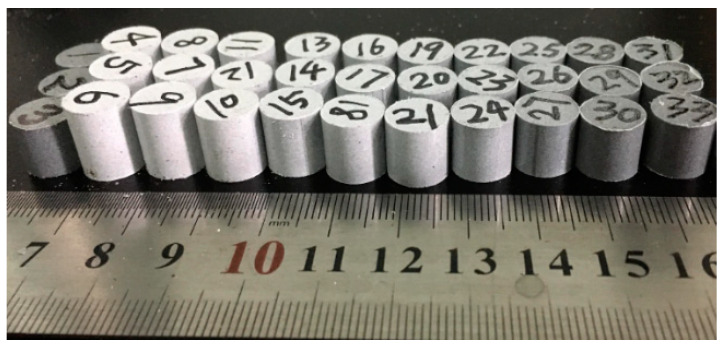
Reactive material samples with different molding pressures.

The PTFE/Al/W component powders were firstly mixed via a dry mixing process in an ambient environment for approximately 24 h. After that, the dried powder mixtures were encapsulated in a rigid Φ10.0 mm cylindrical mandrel with a moving piston. Pressure applied to the reactive mixtures ranged from 5 MPa to 150 MPa, with a dwell time of approximately 1 min at the ambient temperature. The pressed samples were then relaxed at ambient temperature for 24 h. Figure 1 shows parts of the cold-pressed samples. Then, parts of the samples were put into a furnace to undergo a sintering cycle with the protection of an argon atmosphere. The sintering cycle included heating the samples at the rate of 115 °C/h to the final temperature of 365 °C, holding at the final temperature for 2 h, and finally cooling to room temperature at the rate of 115 °C/h.

The reactive material sample during the heating/cooling stage could be assumed as a three-dimensional short cylinder under unsteady heat conduction conditions. From the symmetry of the cylinder, for any reactive material shaft section, in the heating/cooling process, the element of the material temperature was only related to its position and sintering time [16]. A long sintering cycle is a benefit for reducing the temperature gradient in the sample and removing the influence of the heating/cooling process. The heating/cooling rate in our research is 115 °C/h, which is also steadier than the fabrication principle given in the patents. A 2 h sintering time is programmed to ensure sufficient heat exchange between the samples and atmosphere.

Sintering temperature is suggested in the range of 350 °C to 385 °C based on the early patents about sintered fluoropolymer matrix reactive materials [21,22]. The actual temperature peaked at a higher point than the sintering temperature set by using two heating rates (147 °C/h to 1300 °C/h). However, a lower sintering temperature always leads to a brittle property and low strength [15]. In addition, when the sintering temperature exceeded 370 °C, the matrix of the reactive material experienced thermal decomposition [16]. Thus, we selected 365 °C as the sintering temperature considering both the material strength and overshooting temperature.

The samples are relaxed and sintered for the long-term to remove entrapped air and residual stress [16,23]

### 2.2. Drop Weight Test

**Figure 2:**
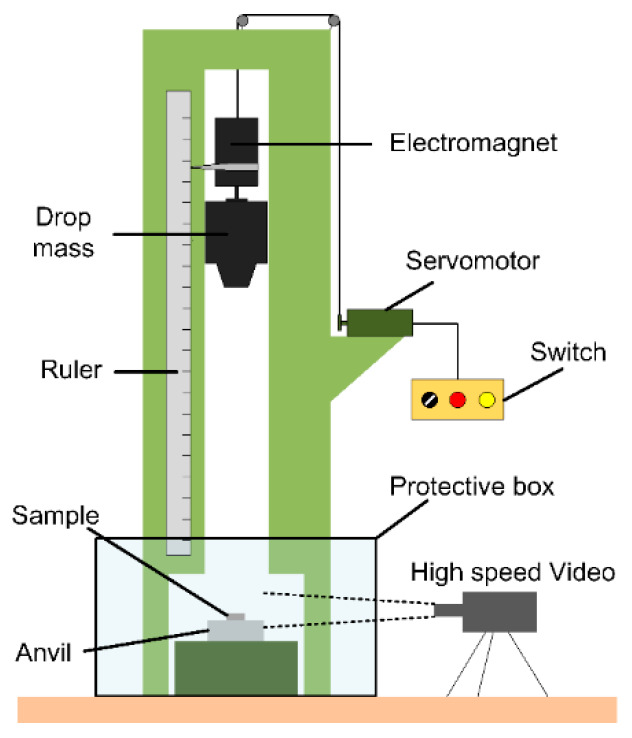
Schematic of the drop weight test apparatus.

In this research, the standard drop weight apparatus was adopted to investigate the sensitivity and impact initiation characteristics of the materials, as illustrated in Figure 2. The tester had a drop mass of 5.0 kg which could be released from a variable height ranging from 0 cm to 200 cm. The rise and release of the drop mass were controlled by an electromagnetic switch. The impact sensitivities of different material samples could be characterized by the characteristic drop height *H*_50_, at which the material had a 50% probability of reaction. The test method by which the 50% point is obtained is an adoption of the well-known “up-and-down” technique [15,24]. The characteristic drop height of impact sensitivity (*H*_50_) can be calculated by Equation (1):

(1) $$H_{50}=H_{0}+\Delta h\left(\frac{\sum iC_{i}}{N}-\frac{1}{2}\right)$$

where *H*_0_ is the lowest height in the test, △*h* is the increment of height, *N* is the number of reaction events among the tests, *i* is the order of the drop height starting from 0, *C_i_* is the number of reaction events under a certain height.

Time sequences of all the impact events were recorded by a Phantom V710 high-speed camera (Vision Research, Inc., Wayne, NJ, USA), with the frame rate, resolution and exposure time were set as 8000 fps, 640 × 480 pixels and 30 µs, respectively.

### 2.3. Characterization

The mesostructure of porous reactive materials, including particle geometry, inner shaping morphology such as pore and cracks, plays a significant role in its impact initiation characteristics. To further observe the mesostructure of the CIPed and HTSed PTFE/Al/W reactive materials, images obtained by backscatter diffraction (BSD) are collected using optical and scanning electron microscopy (SEM, HITACHI S-4800, CamScan, Tokyo, Japan). SEM specimens are prepared by cutting the CIPed and HTSed samples into 1–2 mm thick sections along the axis with a scalpel and then coating with Au. The cross-sections of the specimens are randomly selected for observations with an accelerating voltage of 5 kV.

## 3. Density Homogenization Characteristics

### 3.1. Compactness in Mesoscale

**Figure 3:**
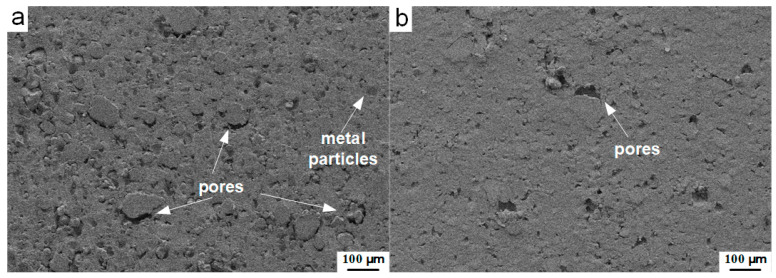
Cross-sectional mesoscale images of the type C cold isostatic pressed (CIPed) PTFE/Al/W samples pressed under different pressures: (**a**) 10 MPa; (**b**) 100 MPa.

Different molding pressures lead to different compactness of the porous samples, resulting in reactive materials with various bulk densities. Figure 3 shows the cross-sectional mesoscale images obtained by backscatter diffraction (BSD) methods with the same magnification times of the type C CIPed samples pressed under the pressure of 10 MPa (Figure 3a) and 100 MPa (Figure 3b).

Under the pressure of 10 MPa, the cross-section of the sample shows a rougher surface, compared with the material pressed under higher pressure (100 MPa). The metal particles could be easily identified through their circular geometry and the interfaces between the particles and the matrix. Dense pores wrapped by matrix and particles could be observed. As the molding pressure is further increased to 100 MPa, the number of pores decreased significantly. The surface of the cross-section appears smoother, while the morphology of the whole mesoscale structure shows a well-compacted characteristic.

**Figure 4:**
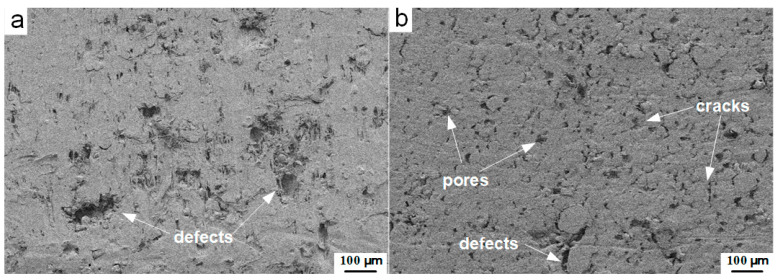
Mesoscale structures of the type C PTFE/Al/W samples pressed under the pressure of 25 MPa: (**a**) hot temperature sintered (HTSed), (**b**) CIPed.

To reveal the effect of the sintering process on the compactness of the material, the HTSed and CIPed type C material pressed under 25 MPa are compared. Figure 4 shows the cross-sections mesoscale images obtained by backscatter diffraction (BSD) methods with the same magnification times of the type C samples pressed under 25 MPa. Despite the structural damage caused by the sectioning process, the obviously densified structure could be observed from the HTSed specimen (Figure 4a). By contrast, a large number of pores, cracks and defects could be found from the loose and porous mesoscale structures of the CIPed sample (Figure 4b). These all demonstrate that the sintering cycle is an effective way to improve the compactness of the material and reduce the defect in the structure. Figure 4b also presents that with the molding pressure rising to 25 MPa, compared with that of 10 MPa in Figure 3a, the cross-section appears flatter and smoother. This further illustrates the importance of molding pressure on the improvement of the compactness of the reactive material.

### 3.2. Bulk Density Homogenization

**Figure 5:**
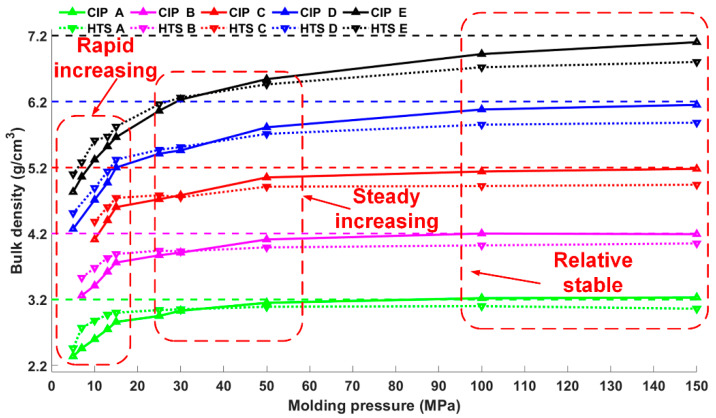
Bulk densities’ increasing tendency of porous PTFE/Al/W reactive materials.

The microstructure of porous reactive material changes during the pressing/sintering process and influences the material bulk density. Figure 5 presents the changes in the bulk densities with the changes of the molding pressures of the five types of CIPed and HTSed PTFE/Al/W materials. Due to the regular geometry of the reactive materials presented in this research, their bulk densities are calculated by dividing its mass by its volume [25]. Basically, with the increase of the molding pressure, the change of the bulk densities would experience three stages: rapid increasing stage, steady increasing stage and stable stage, as demonstrated in Figure 5.

With the increase of molding pressure, the bulk densities of the CIPed and HTSed materials first increase rapidly. In this stage, the HTSed materials show higher bulk densities than the CIPed specimens. The sintering process contributes to the compactness of the mesoscale structures, which should be responsible for the bulk densities increase of the HTSed material.

As the molding pressure increases, bulk densities of the CIPed and HTSed samples continue to increase, until the curves of the CIPed and HTSed materials intersect at a certain molding pressure. However, as the molding pressure further increases, bulk densities of the CIPed samples increase faster, which results in higher bulk densities of the CIPed specimens than the HTSed ones. This should be attributed to the melting-crystallization transformation of the PTFE matrix [16]. Following the steady increasing stage, bulk densities of the two types of specimens remain relatively stable. Materials fabricated by different methods all achieve their TMDs.

Failure strength of reactive material composite with bulk densities 7.96–9.65 g/cm^3^, in which Al and W particle size are the same, range from 25 MPa to 30 MPa [26]. When decreasing the bulk densities lead to low strength, however, the grading particles’ size provide benefits for the strength. Therefore, in the compaction process of reactive materials, 25–30 MPa may form the best material pores and CIPed and HTSed densities are switched.

PTFE/Al/W composite materials apply well to Equation (2) in which the assumption is fitting for analyzing the relation between bulk densities *ρ*_0_ and molding pressures *P* of pressed energetic material composites [27]. The bulk densities of the reactive materials increase in the natural logarithm relationship with the molding pressure like Equation (2).

(2) $$\rho_{0}=a+b\ln P$$

where *a*, *b* are constants involved in the pressing process.

We accordingly fit the bulk densities of the experiment samples results by Equation (2) and found that fitting bulk density results are in good agreement with experiment results. In this way, the fitting curves could provide the relation between bulk densities and molding pressures of porous reactive materials.

**Figure 6:**
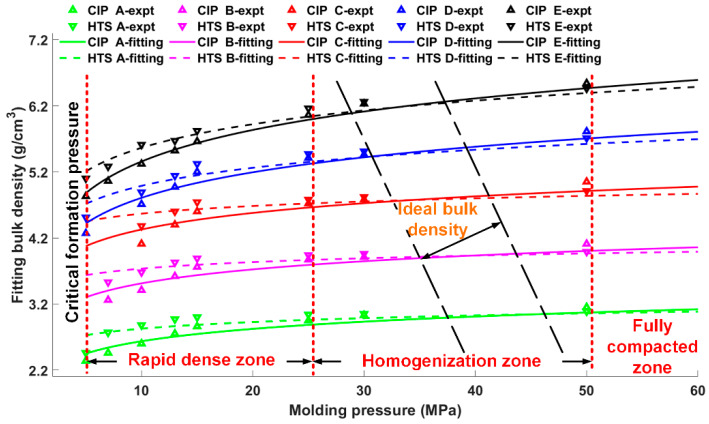
Relation between bulk densities and molding pressures of porous reactive materials.

Further fittings of the bulk densities of CIPed and HTSed reactive materials by Equation (2) with a confidence interval of 95% are expressed as Equation (3) and are presented in Figure 6.

(3) $$\left[\begin{array}{c} \begin{array}{cc} \rho_{\mathrm{CIP}\;A} & \rho_{\mathrm{HTS}\;A} \end{array}\\ \begin{array}{cc} \rho_{\mathrm{CIP}\;B} & \rho_{\mathrm{HTS}\;B} \end{array}\\ \begin{array}{c} \begin{array}{cc} \rho_{\mathrm{CIP}\;C} & \rho_{\mathrm{HTS}\;C} \end{array}\\ \begin{array}{cc} \rho_{\mathrm{CIP}\;D} & \rho_{\mathrm{HTS}\;D} \end{array}\\ \begin{array}{cc} \rho_{\mathrm{CIP}\;E} & \rho_{\mathrm{HTS}\;E} \end{array} \end{array} \end{array}\right]=\left[\begin{array}{c} \begin{array}{cc} 2.015 & 2.494 \end{array}\\ \begin{array}{cc} 2.811 & 3.406 \end{array}\\ \begin{array}{c} \begin{array}{cc} 3.493 & 4.181 \end{array}\\ \begin{array}{cc} 3.522 & 4.080 \end{array}\\ \begin{array}{cc} 3.770 & 4.378 \end{array} \end{array} \end{array}\right]+\left[\begin{array}{c} \begin{array}{cc} 0.2330 & 0.1444 \end{array}\\ \begin{array}{cc} 0.3045 & 0.1428 \end{array}\\ \begin{array}{c} \begin{array}{cc} 0.3621 & 0.1674 \end{array}\\ \begin{array}{cc} 0.5577 & 0.3941 \end{array}\\ \begin{array}{cc} 0.6887 & 0.5147 \end{array} \end{array} \end{array}\right]\ln P$$

Within the pressure range from 5 MPa to 60 MPa, the axial plane could be further divided into three sections, as indicated by the three vertical red lines. Following the critical formation pressure which denotes the minimum molding pressure for the formation of the CIPed samples, the three sections are rapid dense zone, homogenization zone and fully compacted zone. In the rapid dense zone, bulk densities of CIPed and HTSed materials increase markedly. By comparison, bulk densities of the CIPed materials grow more rapidly. With increasing molding pressures in the homogenization zone, the two types of material tend to be the same. Compared with the further changes of bulk densities in the fully compacted zone, the CIPed and HTSed materials achieve the ideal bulk densities.

The pores distributed in reactive materials deform with the compression process and gradually produce stress concentration, resulting in the failure of the matrix around the pore, which may lead to the generation of cracks in the material. In Figure 4b, the unsintered reactive material has significant volume defects under the condition of insufficient compaction. However, the pore size of the material decreases significantly after sintering and the result is identical to Figure 4a.

The changes in molding pressure lead to porosity variation within 10% [28]. As for the pressed fabrication composites, the relative density gradient at different locations exists along the pressing direction [29]. Considering a tiny element of reactive material, the nonlinear bulk density could be considered as changing linearly along the pressing direction. The gradient distribution of forming pressure results in non-uniform energetic powder density changes shown in Equation (4) [27]:

(4) $$\rho_{h}=\rho_{0}-ch$$

where *c* is constant, *h* is the position depth from the pressing surface in the cylindrical sample and *ρ*_h_ is the bulk density of the reactive material element in the position depth of *h*. Density decreases linearly along the pressing direction.

Combining Equation (4), in which reactive materials are applicable to material assumptions, the bulk density change of reactive material is assumed to be linear in the axial direction of the cylinder.

**Figure 7:**
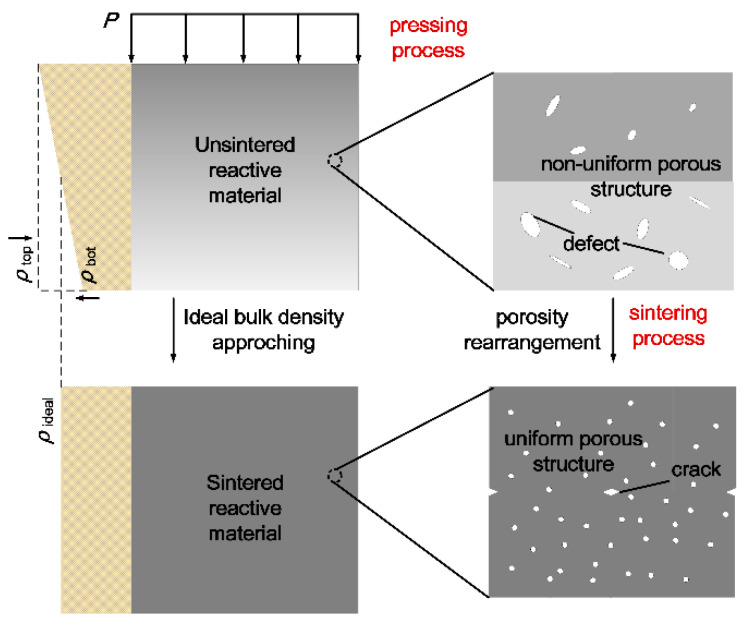
Schematic of gradient molding pressure induced cracks.

To reveal the mechanism for the formation of cracks in Figure 4, two cubic micro-elements of the reactive material with axial pressure distribution are selected for analysis, as shown in Figure 7. As illustrated by Equation (4), gradient molding pressure induces density distribution in pressing direction and causes non-uniform porosity and defects. The non-uniform pressure distribution also results in contractive and expanded deformation around the high- and low-pressure regions. Then, the high-temperature sintering process would result in expansion, extraction and gradient shear stress. When shear stress accumulates, axial and radial cracks are generated in the material. Regardless of the strength of the matrix, thermal residual stress forms between the metal particles during this process [16]. However, the adjacent particles show a trend of convergence during the sintering process. The migration and condensation of particles would result in the rearrangement of discretely distributed tiny gaps in the components system, and thus porosity is rearranged to idealistic in HTSed reactive material.

## 4. Impact Initiation Characteristics

### 4.1. Typical Impact Initiation Phenomenon

**Figure 8:**
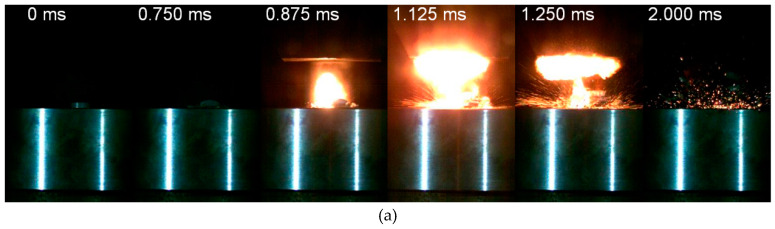

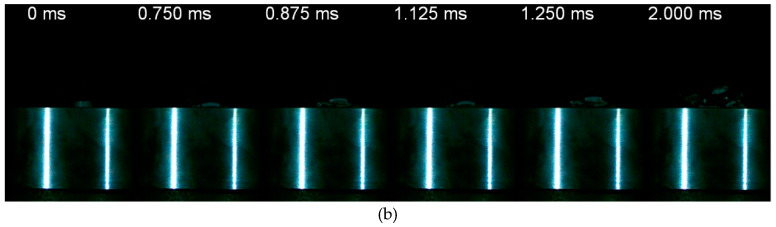
Typical impact response of the PTFE/Al/W reactive materials: (**a**) reacted; (**b**) unreacted.

Generally, impacted by a drop mass at a certain loading rate, the reactive material samples with appropriate molding pressure would be initiated and react violently. Typically, loaded by the drop weight system, the impact and reaction process could be divided into four stages. In the first stage, upon the contact of the top surface of the specimen and the drop mass, the specimen starts to deform elastically and plastically. Quickly after the deformation, in the second stage, some local discrete light and fire which denotes the initiation of the chemical reaction could be observed. As the loading progresses, the discrete fire becomes more intense and brighter gradually, and grows towards the direction where the drop mass bounced back up (Figure 8a, *t* = 0.875 ms). Following this stage, the sample fractures and breaks up. The fire further grows up and the fragments take part in the reaction and sustain the reaction. In this stage, the quick spread and growth of the fire form a mushroom shape, while the reaction of the fragment sprays along the scattering direction (Figure 8a, *t* = 1.250 ms). During the final stage, the reaction gradually stops. Only some remaining fragments react and discrete sparks could be observed (Figure 8a, *t* = 2.000 ms).

PTFE based reactive materials initiated unstably under low-speed impact [15,30,31,32]. That is, the reactive materials could be initiated to form a violent reaction, or it might perform like inert material in identical impact circumstances. In Figure 8b, the same reactive material sample is loaded under the same height as the condition in Figure 8a. The specimen first deforms plastically (Figure 8b, *t* = 0.750 ms) and undergoes fracturing (Figure 8b, *t* = 0.875 ms), then breaks up to blocky pieces splashing outward (Figure 8b, *t* = 2.000 ms). No fire and discrete sparks are observed during the entire process. This impact initiation characteristic could be attributed to the hot spots initiation mechanism of reactive material [1]. For the same reactive material, the internal particles are randomly dispersed. Distribution of the particles, pores/gaps between them have a great influence on the mechanical deformation of the material and the points where the hot spots generally forms [33,34]. This explains the probabilistic impact initiation of the reactive material.

### 4.2. Effect of TMD

**Figure 9:**
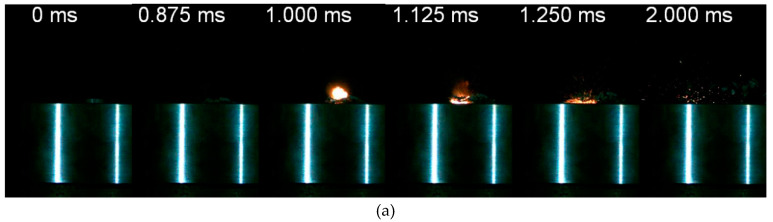

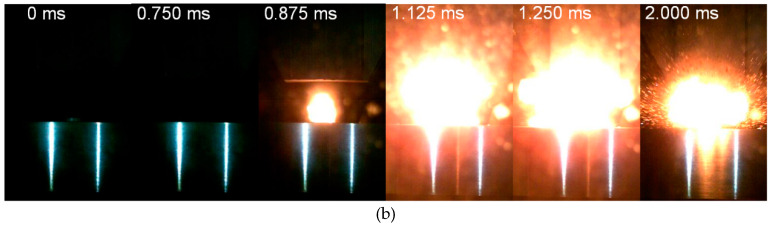
Impact response of HTSed type C and type E reactive material under the same drop height: (**a**) type C; (**b**) type E.

Theoretical maximum density of the PTFE/Al/W reactive material is basically determined by the mass ratios of the components, and plays a vital role in the impact initiation of PTFE/Al/W reactive material [23,35]. Figure 9 compares the impact initiation process of the HTSed type C and type E reactive material molded under the same pressure of 15 MPa and loaded at the same height of 140 cm. During the impact process of the type C material, the first reaction image could be observed at *t* = 1.000 ms. The reaction then attenuated rapidly, the fire burned out along the rebound direction of the drop hammer and the material debris flew along the scattering direction. Then, slight spatter sparks and residual unreacted fragments could be distinguished at *t* = 2.000 ms. By comparison, the initiation of the type E material occurs earlier (*t* = 0.875 ms) and the reaction is more violent (*t* = 1.125~2.000 ms). The reaction first extends rapidly outwards, and then the reaction zone intensifies and forms a continuing glaring reaction cluster. Following this, severe reaction decreases gradually, and the range of the reaction cluster shrinks and sparks sputter around in the complete reaction zone.

From the above images, different impact initiation phenomena could be distinguished, due to the difference in theoretical maximum densities. The main parameter which decides the TMDs is the mass ratio of the W particles. When the material is continuously compressed at a high rate, the internal dislocation and slippage of metal particles occur. The energy will be accumulated gradually due to the formation of densely distributed shear bands. As the content of W or other metal particles increase, the plasticity of the material would decrease [36]. In the process of compression, the materials undergo greater degrees of fracture and fragmentation rather than plastic deformation [37]. Thus, the reaction extent and rate are improved.

### 4.3. Effect of Drop Height

**Figure 10:**
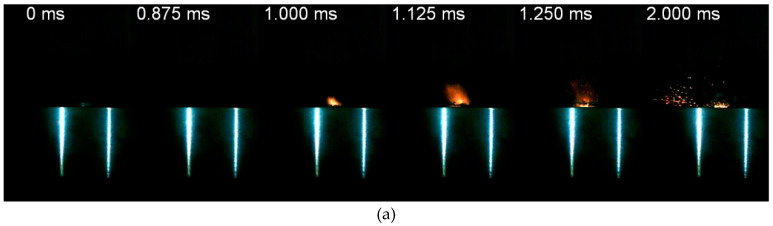

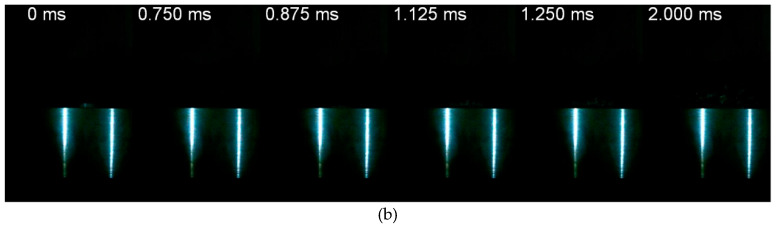
Impact response of HTSed type E reactive material at different drop height: (**a**) *h* = 120 cm; (**b**) *h* = 100 cm.

Compared with Figure 9b, decreased drop height significantly reduces the initiation intensity of the reactive material (Figure 10). Figure 10 presents the HTSed type E reactive material loaded at a height of 120 cm and 100 cm, respectively. Comparing the responding process in Figure 9b and Figure 10, an obvious slow initiation and weak reaction could be observed. When the drop height is 120 cm, the initiation behavior develops similarly to the type C reactive material. The reaction zone of the material enlarges firstly, and then the intensity of the flare is reduced and the reaction is weakened. The reaction only occurs surrounding the material placement area (Figure 10a, *t* = 1.250 ms). Unreacted fragments and spattering activity can be observed while the reaction continues (Figure 10a, *t* = 2.00 ms). Comparatively, when the stimulus is insufficient at low drop height, only an impact and compression process with its resulting fracture and fragmentation of the reactive material sample could be observed. No initiation or reaction would happen, which indicates the existence of an initiation threshold. Only disintegration and fragmentation of the material occurred when the drop height decreased to 100 cm (Figure 10b).

As the falling height of the drop mass increases, the input energy of the drop hammer to the reactive material is merely related to the gravitational potential energy of the hammer [38,39]. Meanwhile, a higher drop height results in a higher impact velocity of the hammer, which leads to higher loading strain rates. This means a higher rate of energy imparted into the material and causes the difference in the initiation and reaction phenomenon.

### 4.4. Effect of Molding Pressure

In Section 3, the effects of molding pressure on the compactness of the material are discussed. It was shown that molding pressure has a significant effect on the mesoscale structures of the material. In consideration of the effect of the mesoscale structure on the formation of hot spots, in this section, the effect of molding pressure on initiation would be discussed.

**Figure 11:**
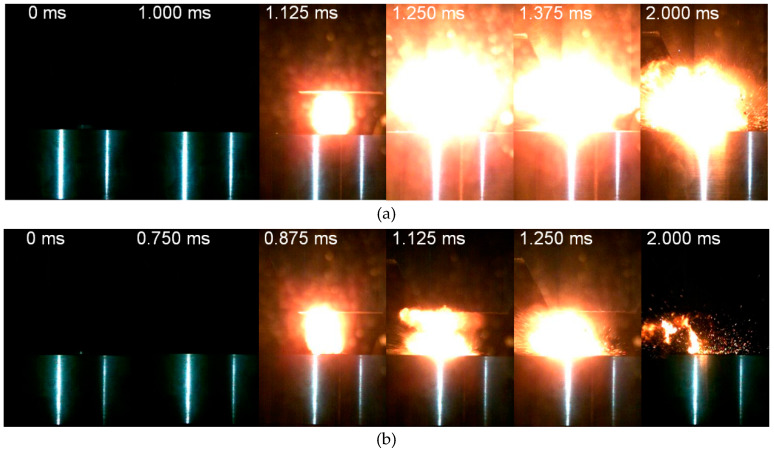

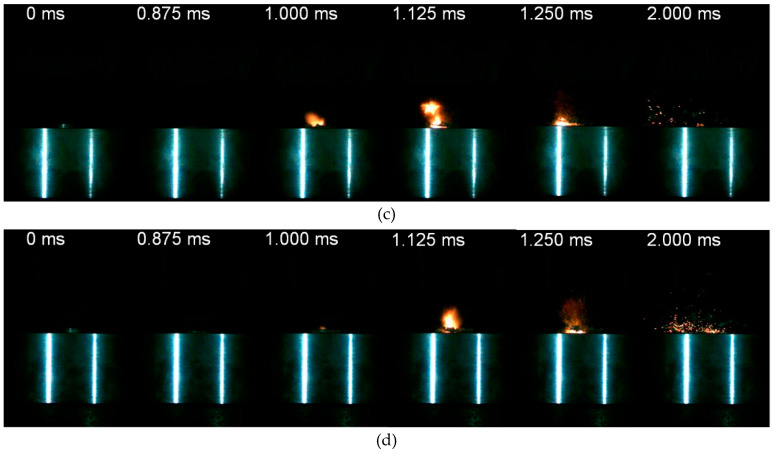
Impact response of the HTSed type E reactive material molded at different pressures: (**a**) 20 MPa; (**b**) 30 MPa; (**c**) 70 MPa; (**d**) 100 MPa.

Figure 11 presents the initiation and reaction process of HTSed type E reactive material molded at pressures of 20 MPa, 30 MPa, 70 MPa and 100 MPa. Comparing the four conditions, the 20 MPa material reacts the most intensively. The flame expands rapidly after initiation and the reaction is slightly weakened in the continuous process. Quite a few unreacted fragments of the reactive material are noticed (Figure 11a, *t* = 2.000 ms). The reacted zone of 30 MPa reactive materials are divided into two parts (Figure 11b, *t* = 0.875~1.125 ms). One part is the flame that grows upwards and turns downwards when it touches the surface of the drop hammer. The other part is the reaction zone formed by the further reacted raw materials. Then, the two reaction zones superimposed to form an enhanced violent reaction area (Figure 11b, *t* = 1.250 ms). However, the subsequent reaction degraded to discontinuous flame and sputtering sparks, and no unreacted material fragments are observed. As the molding pressure increases to 70 and 100 MPa, the intensity of the initiation and reaction decreases significantly (Figure 11c,d). Sputtering sparks associated with unreacted material fragments could be observed at the final moments of the loading process.

During the compression process, the pores in the material would be further homogenized, as shown in Figure 7. The non-uniform density distribution in energetic materials benefits the formation of hot spots where the initiation originates. As the molding pressure increases, the internal porosity decreases and homogenizes gradually. In the following sintering process, the mesoscale porosities would re-accommodate towards homogeneous distribution. Under high-rate impact, hot spots form unsatisfactory and lead to a decreasing trend to initiation probability and reaction intensity.

### 4.5. Impact Sensitivity

**Figure 12:**
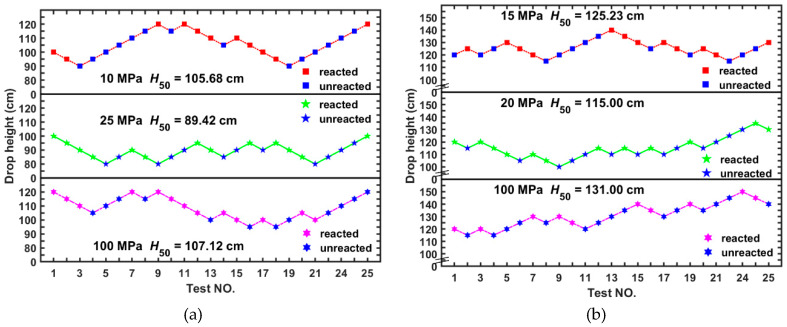
Drop weight test results of the HTSed reactive materials molded under different pressures: (**a**) type C; (**b**) type E.

The effect of molding pressures on the impact sensitivity was studied and compared by the drop weight tests. Results of the HTSed type C and type E samples at three typical molding pressures are depicted in Figure 12. For the type C samples, when the molding pressure is 10 MPa, the characteristic drop height of the initiation is 105.68 cm, while that for the molding pressures of 25 MPa and 100 MPa are 89.42 cm and 107.12 cm, respectively, generally showing first a decreasing and then increasing trend (Figure 12a). For the type E samples, the *H*_50_ changes from 125.23 cm to 115.00 cm and 131.00 cm, showing a similar trend (Figure 12b).

**Figure 13:**
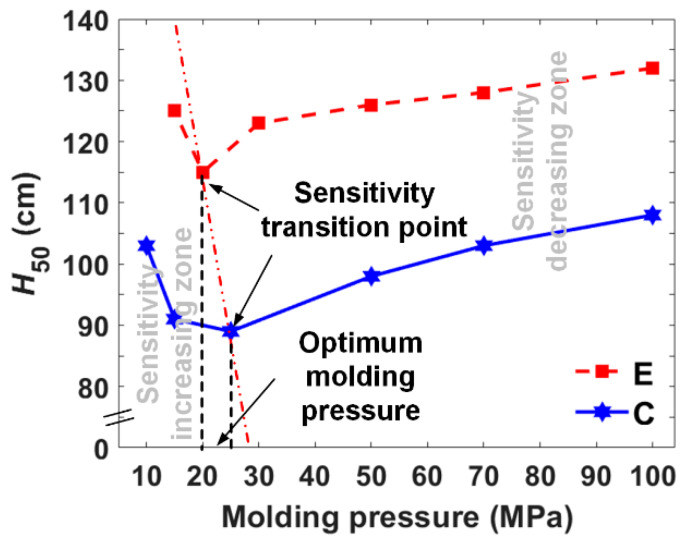
Relationship between the characteristic drop height (*H*_50_) and molding pressure of the type C and type E PTFE/Al/W reactive material.

Incorporating the characteristic drop heights for the two types of materials involving more systematic molding pressures (Figure 13), a more detailed change of the trend could be observed. Generally, the lower the material density is, the higher the impact sensitivity could be achieved, which would be easily explained by the hot spot induced initiation mechanism. Apart from the sensitivity increasing stages, a slightly decreasing trend as the molding pressure increases achieves a stable impact sensitivity with further improvements of the molding pressure.

The axial plane could be divided into two zones: the sensitivity increasing zone and decreasing zone, within which the sensitivities showing increasing and decreasing trends correspondingly. Accordingly, the sensitivity transition points could be identified, which denotes the highest sensitivities for the two types of materials. The molding pressures corresponding to the two highest sensitivities could be defined as the optimum molding pressures. Under this condition, the molding pressure corresponding to a certain impact sensitivity could be determined selectively. The pore distribution and pore size cause sensitivity inflection that drops the height decreases then increases. Bulk densities and impact initiation characteristics are both typical materials properties. The researchers provide a wide range of bulk densities fabricated by various molding pressures, TMDs and relations between material bulk density to its impact initiation characteristics by sharing the same optimum molding pressure.

## 5. Conclusions

The bulk density homogenization and impact initiation characteristic of porous PTFE/Al/W reactive materials are studied. The main conclusions are presented as follows:

(1) Molding pressures lead to a non-uniform bulk density distribution of the porous reactive material. However, the bulk density of porous reactive materials increases logarithmically with the molding pressure and is homogenized towards uniformity during further loading.

(2) Uniform material porosity could be formed in a certain molding pressure range, in which the bulk densities of CIPed and HTSed reactive material achieve stability. Additionally, an ideal bulk density appears in the homogenization zone, which indicates the optimum bulk densities for the CIPed and HTSed material samples.

(3) Theoretical maximum densities, drop heights and molding pressures all have a significant effect on the impact initiation characteristics of the material. Drop heights affect impact initiation characteristics by affecting the amplitude and rate of energy imported into the material system. The theoretical maximum densities and molding pressures play their roles by the degree of mesoscale defect distributions and ratio of Al/W particles.

(4) Sensitivity transition points, correlating the characteristic drop heights to molding pressures, denoting the highest sensitivities, could be identified from the characteristic drop heights for different reactive materials. Ideal bulk densities form by uniform porous structure cause the sensitivity transition points. This would provide a useful guide for the preparation of reactive materials.

## Figures and Tables

**Table 1:** Theoretical maximum densities (TMDs) and corresponding component mass ratios of PTFE/Al/W for multiple tests.

Type	Bulk Density Tests	Drop Weight Tests	TMD (g/cm^3^)	Mass Ratios (wt.%)		
				PTFE	Al	W
A	√	-	3.2	51.01	18.39	30.60
B	√	-	4.2	36.46	13.14	50.40
C	√	√	5.2	27.46	9.91	62.60
D	√	-	6.2	21.39	7.71	70.90
E	√	√	7.2	16.98	6.12	76.90
